# Gastroduodenal artery pseudoaneurysm hemorrhage 1 year after laparoscopic distal gastrectomy: a case report

**DOI:** 10.1186/s40792-020-00802-3

**Published:** 2020-02-18

**Authors:** Aina Kunitomo, Kazunari Misawa, Yozo Sato, Yuichi Ito, Seiji Ito, Takahiro Hosoi, Masataka Okuno, Eiji Higaki, Taihei Oshiro, Seiji Natsume, Takashi Kinoshita, Yoshiki Senda, Tetsuya Abe, Koji Komori, Yoshitaka Inaba, Yasuhiro Shimizu

**Affiliations:** 1 Department of Gastroenterological Surgery, Aichi Cancer Center Hospital, 1-1, Kanokoden, Chikusa-ku, Nagoya City, 464-8681 Japan; 2 Department of Diagnostic & Interventional Radiology, Aichi Cancer Center Hospital, 1-1, Kanokoden, Chikusa-ku, Nagoya City, 464-8681 Japan

**Keywords:** Gastrectomy, Pseudoaneurysm, Delayed bleeding

## Abstract

**Background:**

Postoperative bleeding originating from pseudoaneurysms after radical gastrectomy is not common, but it can be fatal. In particular, delayed bleeding that occurs after the seventh postoperative day is rare.

**Case presentation:**

A 54-year-old man underwent laparoscopic distal gastrectomy, D2 lymph node dissection, and Roux en-Y reconstruction for duodenal neuroendocrine tumors. Drainage was performed for a postoperative pancreatic fistula and abdominal abscess. On the 28th postoperative day, he passed a large amount of bloody stool; therefore, emergency esophagogastroduodenoscopy (EGD) and angiography were performed. However, neither examination demonstrated any bleeding foci or pseudoaneurysm. He was conservatively observed and discharged on the 50th postoperative day. Approximately 1 year after the surgery, he passed a bloody stool and experienced hemorrhagic shock. An EGD revealed exposed blood vessels at the duodenal blind end. His condition was diagnosed as a pseudoaneurysm arising from gastroduodenal artery, which ruptured into the duodenum, based on abdominal contrast-enhanced computed tomography findings. Emergency angiography was performed, and the pseudoaneurysm and artery were successfully embolized.

**Conclusions:**

This case illustrates that there is a possibility of delayed bleeding even 1 year after gastrectomy. Such cases may be serious and require immediate and careful management.

## Background

Although postoperative bleeding after radical gastrectomy for gastric cancer occurs infrequently (2–4% of cases) [1, 2], it is a serious complication because it can be fatal if not properly treated [3]. In particular, delayed bleeding after radical gastrectomy is rare. Li et al. [4] reported that the incidence of delayed bleeding that occurred 7 days postoperatively was 0.9%, whereas that occurred 10 days postoperatively was 0.25% as reported by Song et al. [5]. When we searched for the keywords “gastrectomy” and “delayed bleeding” since 1990 in PubMed [3–9], it was found that the case with the longest time between surgery and bleeding was that of bleeding 90 days postoperatively [7]. We report a case in which an abdominal pseudoaneurysm ruptured into the duodenal blind end approximately 1 year after laparoscopic distal gastrectomy and resulted in massive bleeding. The delayed bleeding after an extremely long asymptomatic time interval between the initial radical gastrectomy and onset of bleeding observed in our patient is extremely rare.

## Case presentation

A 54-year-old man with a body mass index of 43.7 kg/m^2^ (weight, 124.8 kg; height, 169.0 cm) had a medical history of high blood pressure, bronchial asthma, sleep apnea syndrome, and surgery for a right bronchial gangliocytic paraganglioma. He had previously undergone esophagogastroduodenoscopy (EGD) at another hospital for screening examination, without any symptoms and abdominal findings. EGD revealed a tumor in the duodenal bulb; therefore, he was admitted to our hospital for close examination.

**Fig. 1:**
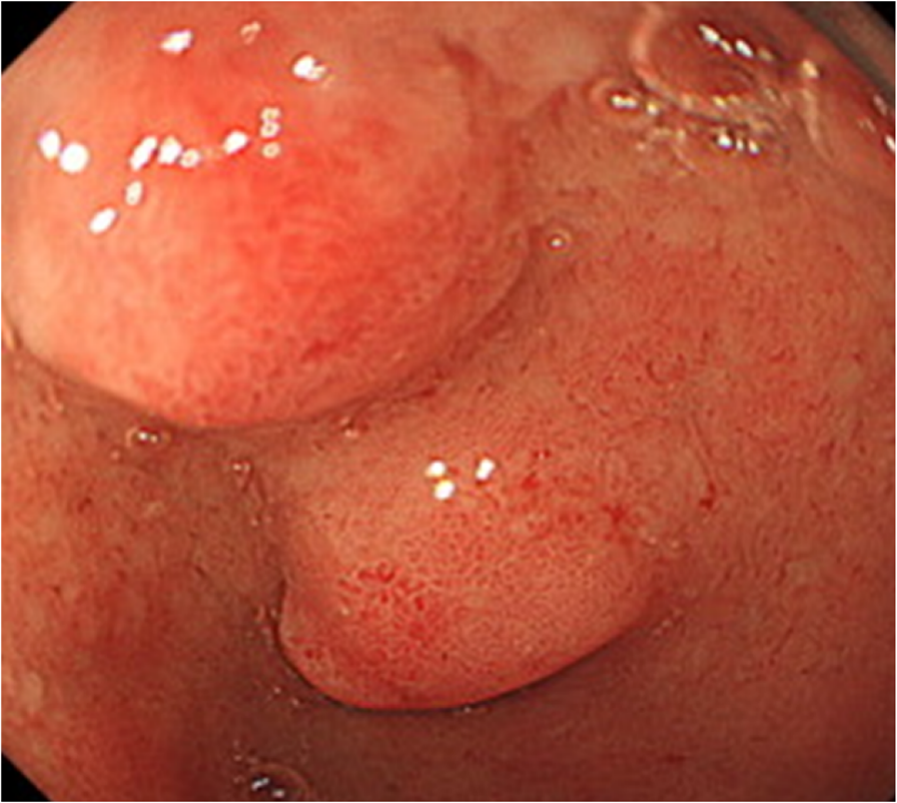
Esophagogastroduodenoscopy (EGD) findings. EGD showing two smooth elevated lesions with a diameter of 10 mm in the anterior wall of the duodenal bulb

EGD revealed two smooth elevated lesions with a diameter of 10 mm in the anterior wall of the duodenal bulb (Fig. 1). A biopsy of the tumors in the anterior wall indicated the presence of neuroendocrine cells. The tumors were diagnosed as duodenal neuroendocrine tumors (NET). Abdominal plain computed tomography (CT) revealed no lesions, apparent enlarged lymph nodes, or distant metastasis. Endoscopic ultrasonography showed that the lesions were hypoechoic masses primarily comprising the third layer having an unclear border with the fourth layer. Because this finding suggested that the tumors had invaded the muscle layer, we decided that surgical resection was necessary. Therefore, laparoscopic distal gastrectomy with lymph node dissection and Roux en-Y reconstruction was performed. The extent of lymph node dissection was D1+ with No.12a lymph node because the tumors were located in the duodenum bulb. Intraoperative findings revealed that the tumors were not exposed to the serosal surface. The surgery was performed in the same way as usual for gastric cancer. The duodenum was transected using linear stapling device, and the stump was reinforced with serosal muscle suturing. A curative resection was performed without intraoperative complications. On the basis of pathological findings and immunostaining, the diagnosis was NET, which was classified as NET G1 according to World Health Organization (WHO) classification 2010 [10].

**Fig. 2:**
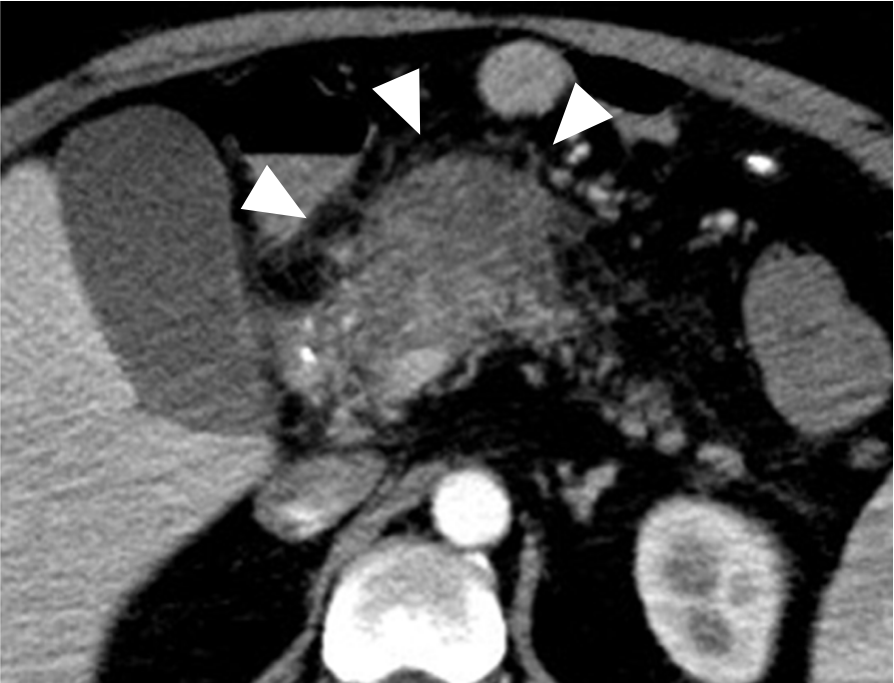
Abdominal plain computed tomography (CT) findings. Plain CT on POD5 showing fluid collection in the anterior cavity of the pancreatic head (arrow)

From the physical findings and laboratory test results, the postoperative course up to postoperative day (POD) 3 was uneventful. The drain amylase level at POD1 was as low as 157 U/L; he started eating on POD3. He had a fever on POD5; hence, the meal was stopped, and a course of antibiotics was started. However, the fever was not improved, so CT was performed on POD8. Abdominal plain CT showed fluid collection in the anterior cavity of the pancreatic head (Fig. 2). Consequently, CT-guided drainage of the collected fluid was performed. The contrast radiography through the drainage tube showed that there was no fistula between the fluid and gastrointestinal tract and no obvious anastomotic leakage. The amylase level of the drainage fluid was high at 4667 U/L, which suggested that the peripancreatic fluid collection was caused by pancreatic fistula and poor drainage. The drainage was continued, and the fluid cavity tended to shrink.

**Fig. 3:**
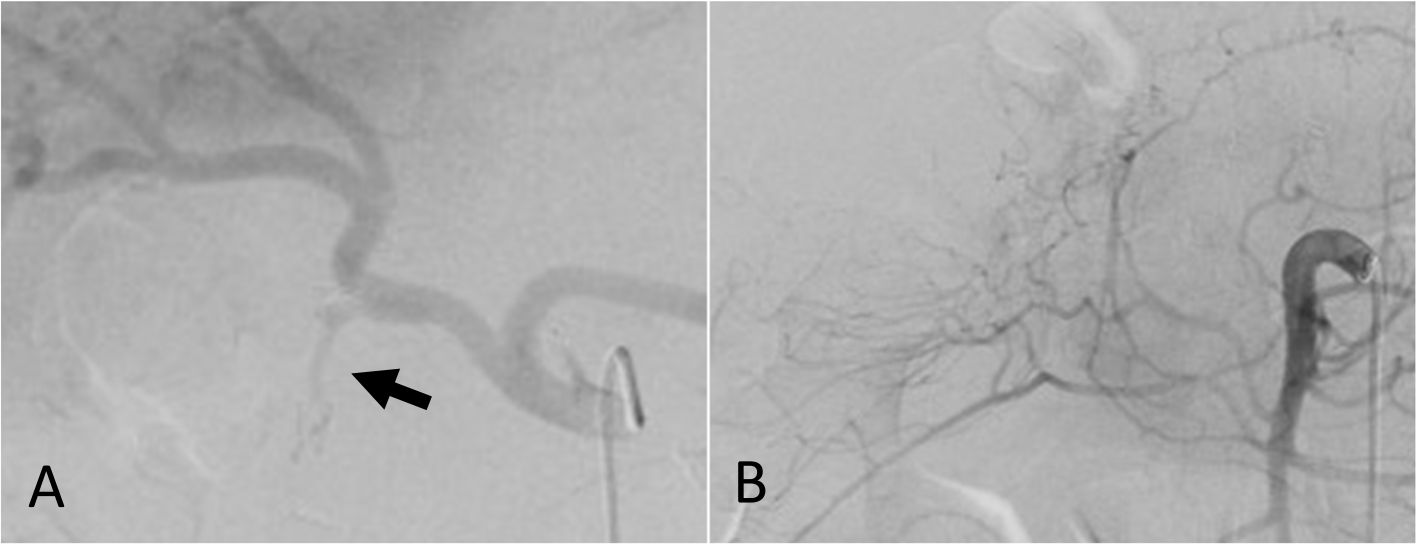
Angiography findings. Angiogram revealing no apparent pseudoaneurysm or extravasation from the celiac artery, splenic artery, and gastroduodenal artery (arrow) and their branches (**a**) as well as inferior pancreaticoduodenal artery branching from the superior mesenteric artery (**b**)

On POD28, he passed a large amount of blood stool. His vital signs were stable. Abdominal contrast-enhanced CT showed a small amount of fluid collection on the anterior side of the pancreatic head but no apparent extravasation findings. Although the CT imaging showed no evidence of abdominal bleeding or pseudoaneurysm, bleeding from a pseudoaneurysm was suspected because of the clinical findings; therefore, an emergency angiography was performed. We performed angiography of the celiac artery, splenic artery, and gastroduodenal artery as well as their branches that perfused the pancreas and the inferior pancreaticoduodenal artery branching from the superior mesenteric artery; however, no apparent pseudoaneurysm or extravasation was found. Therefore, no action other than observation was performed (Fig 3a, b).

EGD showed no bleeding from the gastrojejunal anastomosis site, jejunojejunostomy, and duodenal blind end. Colonoscopy also revealed no bleeding foci. We could not detect the source of bleeding, and there was no blood stool subsequently. The meal was restarted on POD34. Abdominal contrast-enhanced CT on POD36 showed no hemorrhage or pseudoaneurysm, and the abscess had disappeared. Therefore, on the same day, we removed the drainage tube. He was discharged on POD50.

A plain CT 6 months after the surgery showed no abnormal findings and no pseudoaneurysm. Approximately 1 year after the initial surgery (POD350), he passed a bloody stool and was admitted to a local hospital after experiencing hemorrhagic shock. Laboratory tests revealed severe anemia with a hemoglobin level of 4.5 g/dL; therefore, blood transfusion was performed. Because colorectal bleeding was suspected, a colonoscopy was also performed; however, the bleeding source was not detected. A few days later, he again experienced a bloody stool and subsequently underwent EGD. An ulcer with pulsating exposed blood vessels near the duodenal blind end was observed. He was diagnosed as having a hemorrhage from a delayed pseudoaneurysm associated with surgery and was transferred to our hospital.

**Fig. 4:**
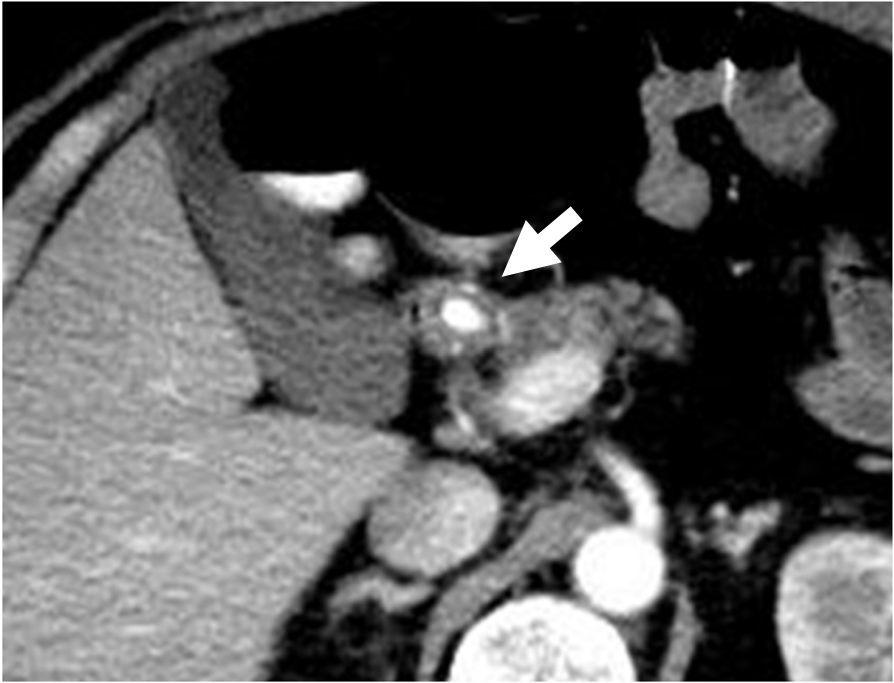
Abdominal dynamic contrast-enhanced computed tomography (CT) findings. Dynamic CT showing a pseudoaneurysm arising from the proximal gastroduodenal artery (arrow)

**Fig. 5:**
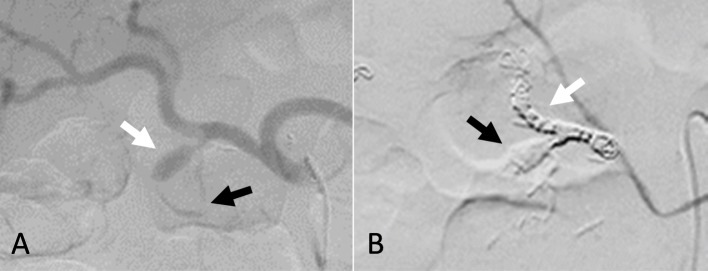
Angiography findings. Angiogram (**a**) showing the pseudoaneurysm developed from the proximal gastroduodenal artery (white arrow) and the posterior superior pancreaticoduodenal artery branched from the pseudoaneurysm (black arrow). Angiogram after transcatheter arterial embolization (**b**) showing the complete hemostasis of the pseudoaneurysm with coils (white arrow) and N-butyl-2-cyanoacrylate (black arrow)

Although his vital signs were stable, laboratory tests showed a hemoglobin level of 7.1 g/dL. Abdominal contrast-enhanced CT revealed a pseudoaneurysm arising from the proximal gastroduodenal artery (GDA), extending into the duodenal wall (Fig. 4). Emergency angiography was performed, which revealed a pseudoaneurysm that had developed from the proximal GDA, as demonstrated in the CT findings (Fig. 5a). Transcatheter arterial embolization (TAE) was successfully performed with the isolation and packing technique. First, the proper hepatic artery distal to the pseudoaneurysm was embolized by using coils. Second, the pseudoaneurysm and posterior superior pancreaticoduodenal artery branching from the pseudoaneurysm were embolized with N-butyl-2-cyanoacrylate. Finally, the common hepatic artery proximal to the pseudoaneurysm was also embolized by using coils (Fig. 5b). Laboratory tests performed 1 day after TAE showed no evidence of liver failure. No clinical signs of rebleeding were observed during the course, and he was discharged on the seventh day after TAE. Subsequently, abdominal contrast-enhanced CT was performed twice approximately 3 and 9 months after TAE, and there were no findings, such as pseudoaneurysm recurrence, and no symptoms suggesting rebleeding.

## Discussion

We reported intraluminal bleeding due to rupture of a pseudoaneurysm that developed 11 months after radical gastrectomy. The pseudoaneurysm was diagnosed by EGD and abdominal contrast-enhanced CT. Emergency angiography was performed, and the pseudoaneurysm and artery were successfully embolized. To our best knowledge, the time from surgery to bleeding, i.e., delayed bleeding after radical gastrectomy, was the longest in our patient.

Postoperative delayed bleeding is mostly caused by rupture of a pseudoaneurysm [11]. Pseudoaneurysm formation is caused by weakness in the arterial wall owing to skeletonization after aggressive lymphadenectomy [9, 11]. An intra-abdominal abscess due to anastomotic leakage also causes erosion of the arterial wall because the vessel wall becomes exposed to enteric, pancreatic, and/or bile juice [5]. In addition, pancreatic juice exposure by pancreatic fistula erodes the arterial wall, resulting in the formation of a pseudoaneurysm [12]. In our patient, there was no apparent anastomotic leakage, but pancreatic fistula and intra-abdominal abscess occurred after the surgery; thus, we assumed that there was pancreatic juice exposure to the arterial wall that may be weakened by lymphadenectomy. Contrast-enhanced CT and angiography performed on POD28 revealed no pseudoaneurysm, and the cause of initial bleeding was not clear. However, because of the clinical course and the fact that the second bleeding was owing to a pseudoaneurysm that developed from GDA, which existed in the area of the lymphadenectomy and postoperative abdominal abscess, the first and second gastrointestinal bleeding were believed to be influenced by pancreatic fistula and abdominal abscess.

Postoperative intraluminal bleeding is usually detected by endoscopy, which is useful for diagnosing anastomotic hemorrhage, although it often fails to determine the bleeding foci in patients with intraluminal bleeding that originated from a pseudoaneurysm [11]. CT angiography is a better technique for detecting bleeding from a pseudoaneurysm.

TAE is the most useful procedure for the treatment of bleeding from pseudoaneurysm [13]. It is suitable as a first-line measure for the management of rupture of pseudoaneurysm because it can determine the exact location of the bleeding [5, 14]. In addition, it is better to manage delayed bleeding for preventing high-risk emergency operative intervention [11, 15, 16]. In our patient, TAE, which is effective for hemostasis, was selected because the bleeding foci were expected to be difficult to reach surgically because of the inflammatory changes after pancreatic fistula, postoperative adhesion, and obesity.

In our patient, no symptoms, such as gastrointestinal bleeding, were observed between the time of the first hospital discharge and onset of the second bleeding. During that period, postoperative follow-up CT for duodenal NET was performed 6 months after the surgery for regular follow-up, but it was plain CT because he had bronchial asthma. Patients with bronchial asthma have a risk of an acute reaction to the iodine-based contrast agent; therefore, contrast-enhanced CT should be avoided for patients without any symptoms or findings. When necessary, steroid premedication is recommended to prevent an acute reaction [17]. In our patient, a pseudoaneurysm may have already existed at 6 months after the surgery, and the pseudoaneurysm could have been detected if contrast-enhanced CT with steroid premedication had been performed. From these experiences, we consider that contrast-enhanced CT should be performed instead of plain CT for regular follow-up within approximately 1 year after radical gastrectomy; this is considering the possibility of postoperative pseudoaneurysm if postoperative complications, such as pancreatic fistula, abdominal abscess, or particularly gastrointestinal bleeding, occurred.

## Conclusion

We reported a rare case of delayed bleeding after radical gastrectomy involving a long asymptomatic period. In cases in which postoperative complications, particularly pancreatic fistula and subsequent intra-abdominal abscess, have occurred, the possibility of delayed bleeding related to a pseudoaneurysm should be strongly considered even if a long time has passed after the surgery. It is critical to perform rapid diagnosis and proper treatment in such cases as the bleeding can be serious.

## Data Availability

Not applicable.

## Abbreviations

CT: Computed tomography
EGD: Esophagogastroduodenoscopy
GDA: Gastroduodenal artery
NET: Neuroendocrine tumor
POD: Postoperative day
TAE: Transcatheter arterial embolization
WHO: World Health Organization
